# Rearing the Maize Weevil, *Sitophilus zeamais*, on an Artificial Maize—Cassava Diet

**DOI:** 10.1673/031.012.6901

**Published:** 2012-06-11

**Authors:** James Adebayo Ojo, Adebayo Amos Omoloye

**Affiliations:** ^1^ Department of Crop Production and Forest Resource Management, Kwara State University, Malete, Kwara State, Nigeria; ^2^ Department of Crop Protection and Environmental Biology, University of Ibadan, Nigeria

**Keywords:** amino acid supplements, pellets, polyphagous, suitable, progeny

## Abstract

Dry artificial diet pellets prepared with maize, cassava chips, and amino acid supplements (lysine and methionine) were evaluated for mass culture of *Sitophilus zeamais* Motschulsky (Coleoptera: Curculionidae), a highly polyphagous pest of many stored grains. Evaluation was done in the laboratory at temperature 26 ± 2 °C, 60–70% RH, 12:12 L:D photoperiod. The artificial diet was compounded from different proportions of maize (M) variety TZPB-SW-R, cassava (C) variety TMS-2110, and amino acid supplements, and was pelletized into 6 mm diameter pellets on which five pairs of one—day—old *S. zeamais* were bioassayed. The diet M_9_C_1_ (90% M and 10% C) was the most suitable diet with comparatively shorter developmental period (34.8 days) and the highest F_1_ emergence of progeny (145.4) compared to the control, M_10_C_0_ (100% M and 0% C).

## Introduction

The development of artificial diet for many insects has contributed immensely to the study and control of insect pests, especially in the areas of entomology, genetics, ecology, and physiology, where regular and dependable supplies of high quality insects are required in adequate numbers and at specific periods (Fortes et al. 2006) for bioassays and related studies. Numerous artificial diets have been described for phytophagous insects, especially for pest species of the Noctuidae and Curculionidae families (Singh 1977; Wheeler and Zahniser 2001). Such diets have been valuable for mass culturing other economically important insects (Cohen 2001; Castane and Zapata 2005; Ahmed et al. 1998). However, there is paucity of information on the mass culture of *S. zeamais* using artificial diet made from maize—cassava that will be adaptable in insect science investigations in tropical countries such as Nigeria.

Insects are major post—harvest pests of crops both at the farmer and consumer level in the tropics (Lale and Ofuya 2001; Adedire 2003). The maize weevil, *Sitophilus zeamais* Motschulsky (Coleoptera: Curculionidae), is a primary field to store pest (Adedire 2001) that starts to infest the ripening maize crop in the field when the grain moisture content is still 50–55%. Thus, when farmers harvest the maize crop 6–8 weeks later, the weevil has already completed one generation and has laid eggs for the second generation (De Lima 1979). Reports from Africa (Hill and Waller 1990) also confirm field—to—store infestation by *S. zeamais.* Additionally, it is a secondary pest of several other crops such as rice, sorghum, yam products, and cassava flour in storage (Nwanna 1993). Post—harvest losses to storage insect pests such as *S. zeamais* have been recognized as an increasingly important constraint to maize production in Africa (Markham et al. 1994). Its heavy infestation may cause weight losses of as much as 30– 40% of produce (CABI 2005). Adult weevils and larvae feed on undamaged grains and reduce them to powdery form (Adedire 2001).

The need to study and control insect pests has contributed to the development of artificial diets. Therefore this study seeks to mass— culture *S. zeamais* on a maize—cassava artificial diet (a non—holidic diet) to test its suitability as a viable diet. In Nigeria, wheat is imported, which makes it very expensive and not readily available. Maize and cassava are commonly grown in many African countries; thus, the development of a maize—cassava diet would be practical and have direct application for use in similar research in Nigeria and other parts of Africa.

## Materials and Methods

### *Sitophilus zeamais* culture

The initial stock of *S. zeamais* used for the experiment was obtained from the Entomology Research Laboratory of the Department of Crop Protection and Environmental Biology, University of Ibadan, Nigeria. Maize grains (200 g) were put in 2 kg capacity kilner jar to which 10 pairs of *S. zeamais* were introduced for oviposition for one week in the laboratory. The set up was replicated six times and samples were observed daily until emergence of F_1_ progenies. The experimental conditions were 60 ± 10% RH, 26 ± 2 °C, and 12:12 L:D.

### Experimental materials used

Cassava chips (TMS-2110 variety) and maize grains (variety TZPB-SW-R) were obtained from the Seed Storage Section, Institute of Agricultural Research and Training (IAR&T), Moor Plantation, Ibadan. The amino acid supplement, lysine and DL-methionine, were sourced from the seed store of Adom Agroservice, Ibadan, Nigeria.

### Determination of the nutritional content of maize—cassava diet

Prior to diet formulation, the nutritional content (primary metabolites and minerals) were determined following the analytical procedures described by AOAC (1990). These form the basis for provisional admixtures of maize—cassava—amino acid supplement diets formulated (Table 2). Each artificial diet treatment was also evaluated using the method of AOAC (1990).

### Artificial diet preparation

Dried cassava chips and maize were fine— ground separately using a lister 5 HSP grinding mill. These were admixed in varying proportions to form 14 different diets (Table 2) on which 100 mL of distilled water was added to a 250 g sample lot of each diet group and admixed thoroughly to form a semi—thick homogenized paste. The paste was then pelletized (6 mm diameter, 1.5 cm length). Pellets were then oven—dried at 105 °C to 13% moisture content.

### Performance of *S. zeamais* on artificial diets

A 20 g sample of each artificial diet pellet group was weighed into a 15 cm diameter Petri dish in five replicates. Five pairs of one— day—old *S. zeamais* were introduced to each replicate following standard procedure (Odeyemi and Daramola 2000). The weevils were sexed by examining the rostrum and abdominal shape of the insects. The rostrum of the male *Sitophilus* is rough, distinctly shorter and wider than that of the female, while the rostrum of the female is smooth, shiny, distinctly longer and narrower than that of the male. The weevils were allowed to mate and oviposit on the diets for seven days, after which they were removed. Another five replicates of each treatment without infestation by *S. zeamais* were set up to monitor moisture content of the diet. Both the infested and uninfested lots were arranged in the laboratory in a completely randomized design. The experimental set up was monitored daily until emergence of F_1_ progenies. The following data were collected: (i) developmental period, (ii) emergence of F_1_ progenies, (iii) weights of F_1_ progenies at emergence, and (iv) sex ratio.

### Statistical analysis

Data were analyzed using analysis of variance (ANOVA), and where significant, means were separated using Tukey's HSD test (*p* < 0.05). Data on emergence of F_1_ progenies, weight at emergence, and developmental period were transformed using square root transformation (x + 0.05)^1/2^ (Little and Hills 1978). Sex ratio was determined following techniques described by Halstead (1963) and Adedire (2001).

## Results

### Emergence, weight, and developmental period of F_1_ progenies of *S. zeamais* raised on artificial diet pellets

Table 1 shows the nutrient content of maize and cassava used in the study; there was little difference in the protein and starch content of the two ingredients. Table 4 shows the effect of artificial diet pellets on the developmental period, mean number emerged, and weight of F_1_ progenies raised on the artificial diet pellets after five weeks. Except for diet M9C1 that had a comparatively shorter developmental period (34.8 days) than the control (M_10_C_0_, 35.6 days), diet M_4_C_1_ (36.2 days), and diet M_7_C_3_ (36.6 days), which were not significantly different from the control, developmental periods were significantly longer (*p* < 0.05) on other artificial diet pellets compared to the control (Table 4).

Similarly, mean emergence of F_1_ progeny was highest (*p* > 0.05) on artificial diet pellet M_9_C_1_ (145) compared to the control (130). Although comparatively lower, the mean number of progeny that emerged on diet M_4_C_1_ was not significantly different from the number that emerged on the control diet (M10C0) (Table 4). The mean body weight of F_1_ progenies from the artificial diet pellets differed significantly from 1.86 mg in CLMe_0.5_ to 2.34 mg in M_10_C_0_ (Table 4). Although slightly higher, mean body weight of F_1_ progeny were not significantly different from the control M_10_C_0_ (2.34 mg) and M_9_C_1_ (2.22 mg).

### Effect of consumption of AD pellets on sex ratio of F_1_ progeny

Significant differences occurred in the sex ratios of weevils that emerged from the AD pellets (Table 5). The sex ratio of F_1_ progeny emerged from all diets conformed to a 2:1 sex ratio in favor of the females (χ^2^ = 14.84: *p* < 0.05).

### Mineral and vitamin composition of artificial diet (AD) pellets formulated

The mineral and vitamin composition of AD pellets are presented in Table 6. Percentage protein ranged from lowest (2.06% in M_0_C_10_) to highest in diet M_10_C_0_ (3.18 %) and diet M_9_C_1_ (3.07 %). Other components also varied: fat (0.43–0.57%), ash (2.08– 2.22%), fiber (0.43–0.57 %), sugar (4.3– 5.8%), and starch (78.95–80.5%). Vitamin C content of all the diets also ranged from 1.88–2.02 mg. Phosphorus was the highest occurring element in all diets, being lowest in diet M_10_C_0_ (251 mg) followed by diet M_9_C_1_ (267.3 mg) and diet CLMe_0.25_ (389.1 mg) (Table 6).

## Discussion

This study shows that *S. zeamais* could be reared in the laboratory on a non—holidic diet, making it relatively easy to mass culture *S. zeamais* for research and other pest management intents and purposes. Other coleopterans such as *Oxyops vitiosa* have been reared on artificial diets made from a mixture of *Melaleuca quinquenervia* leaves, corn starch, and casein (Wheeler and Zahniser 2001), and *Rhynchophorus ferrugineus* was reared on artificial diets made from a mixture of oat, coconut cake, yeast, and sugarcane fibers (Weissling and Davis 1995).

In this study, diet M_9_C_1_, which contained 90% whole maize and 10% cassava, was the most suitable diet for mass culture of this insect. The developmental period (34.8 days) was faster by about 10% compared to the control (M_10_C_0_). Number of F_1_ progeny emerged was also significantly higher (145.4) than the control. Similarly, the mean body weight of F_1_ progenies was not significantly different from the control. All these might be a result of its adequate nutritional quality. Despite the fact that there was not much difference in the protein and starch content of the ingredients used in the diet formulation, diet M_9_C_1_ proved to be the best. This could be because nutrients present in the diet were in the right proportion, as insects reared on plant tissue as well as artificial diet might not develop because the diet's secondary chemistry and/or lower nutritional value might inhibit their optimal development (Blanco et al. 2008). It could also be the result of efficient net food utilization of diet M_9_C_1_ by the weevils.

Significantly longer developmental period (adult days to emergence), low number of F_1_ progeny, with lowest adult body weight of F_1_ progeny recorded on diets M_3_C_7_, M_1_C_4_, and CLMe_0.5_, and no emergence on diets M_1_C_9_, M_0_C_10_, CLMe_1_, and CLMe_0.5_ suggest that they were not suitable for development and mass culture of *S. zeamais.* However, it was observed that adult *S. zeamais* feeding on diets CLMe_1_, CLMe_0.5_, and CLMe_0.25_ had significant weight gains, though this did not affect the emergence of F_1_ progenies of *S. zeamais.* Our observation of longer developmental periods and low adult body weight agrees with findings of Fortes et al. (2006), Panizzi et al. (1991, 2001), Parra (1991), and Panizzi and Rossini (1987), who reared *Nezara viridula* and *Euschistus heros* on a dry artificial diet. They reported that a longer developmental period and low adult body weight correlated with the inadequate nutritional quality of the diets, which gives an indication of the nutritional unsuitability of the tested diets. Additionally, Coudron et al. (2005) reported that longer development times and lower fecundity were observed when *Podisus maculiventris* was reared on zoophytophagous artificial diet. Dietary phosphorus has been shown to affect growth rate and body size (Perkins et al. 2004), population density, reproduction, and survival (Popp et al. 1989). This finding can be compared with that of Baker (1974), who reared *S. oryzae* on an improved casein diet but observed that the weevils failed to develop. This showed that casein was not an optimal source of amino acid for *S. oryzae*, as found in our study where the amino acid supplements (methionine and lysine) did not have any effect on the cassava diets.

The mean development period observed in the diets M_10_C_0_, M_9_C_1_, M_4_C_1_, and M_7_C_3_ agrees with the findings of Haines (1991), who reported that the mean development period of *S. zeamais* at 27 °C and 70% RH varied from 31–37 days, and that of Rees (1996), who reported that the developmental periods takes about 35 days under optimal conditions. Also, the shortest days to emergence (34.8) recorded on diet M_9_C_1_ was longer than what Baker and Mabie (1973) observed when *S. granarius* was raised on natural and meridic diets (wheat, corn, and rice flours, and casein— starch/glucose based meridic diets); they recorded 25.5 and 26.5 days to emergence for female and male, respectively. Furthermore, the mean number of F_1_ progeny of 145.4, 130.8, and 124.2 on M_9_C_1_, M_10_C_0_, and M_4_C_1_ diets, respectively, support the findings of Haines (1991), who reported that the major primary pests of stored grains such as *Sitophilus* spp. are able to increase in number under optimal temperature and moisture conditions by as much as 100 times in each generation on favorable diets, although their development may depend on the kind of stored grain being fed on.

The results of our study have also shown that the sex ratios of F_1_ progeny significantly differ from each other, with a female—to—male ratio of 2:1. This finding did not agree with Danho et al. (2002), who reported that the sex ratios of F_1_ progeny did not differ significantly from each other, even though there were more females than males. This finding also disagreed with Fortes et al. (2006) and Abbasi et al. (2007), who reported a ratio of 1:1 when *Helicoverpa armigera* was reared on a tapioca—based artificial diet.

Our study shows that it is possible to rear *S. zeamais* on non—holidic artificial diets of cassava—maize fortified with synthetic amino acids. Also, the artificial diet pellet prepared and formulated from these locally sourced Nigerian crops is feasible for the mass culture of *S. zeamais*, and the formulated artificial diet pellets M_9_C_1_ is suitable for the mass culture of *S. zeamais* in the laboratory under tropical Nigerian conditions.

**Table 1.**
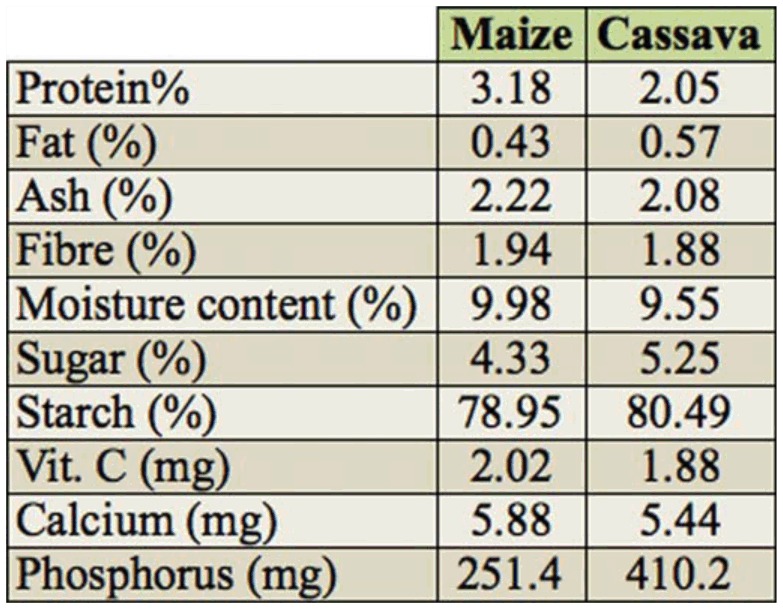
Mineral and vitamin content of maize and cassava chips used in the study, (analysis per 100 g).

**Table 2.**
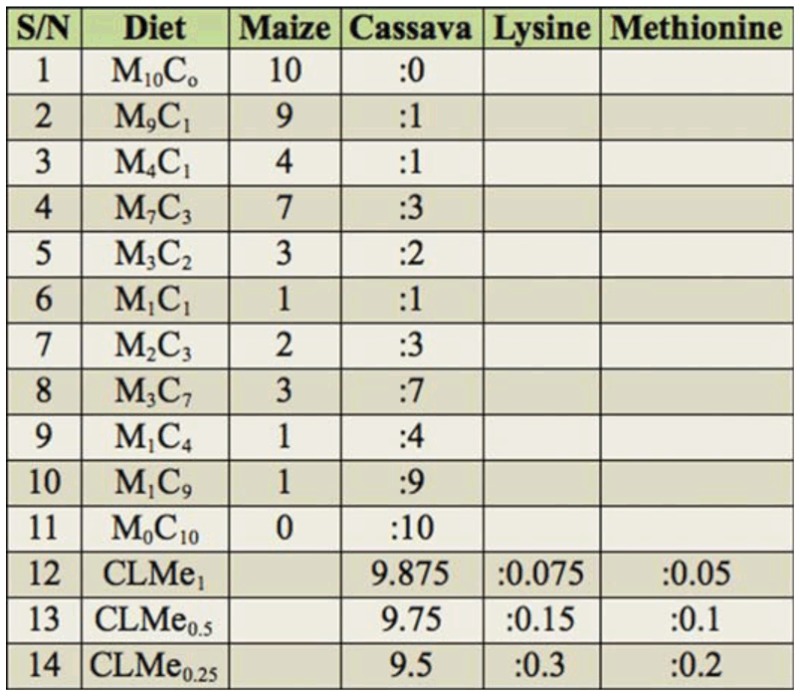
Composition of artificial diets.

**Table 3.**
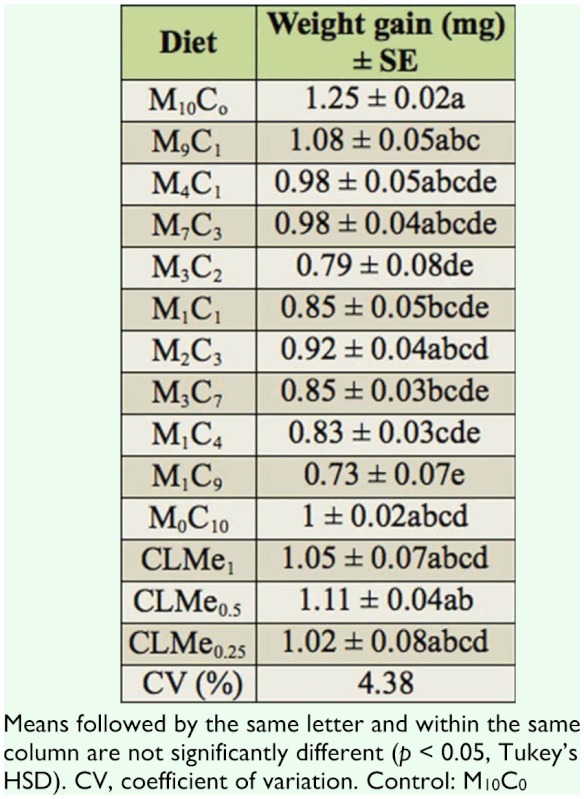
Weight gain by *Sitophilus zeamais* after consumption of AD pellets (mg) ± SE.

**Table 4.**
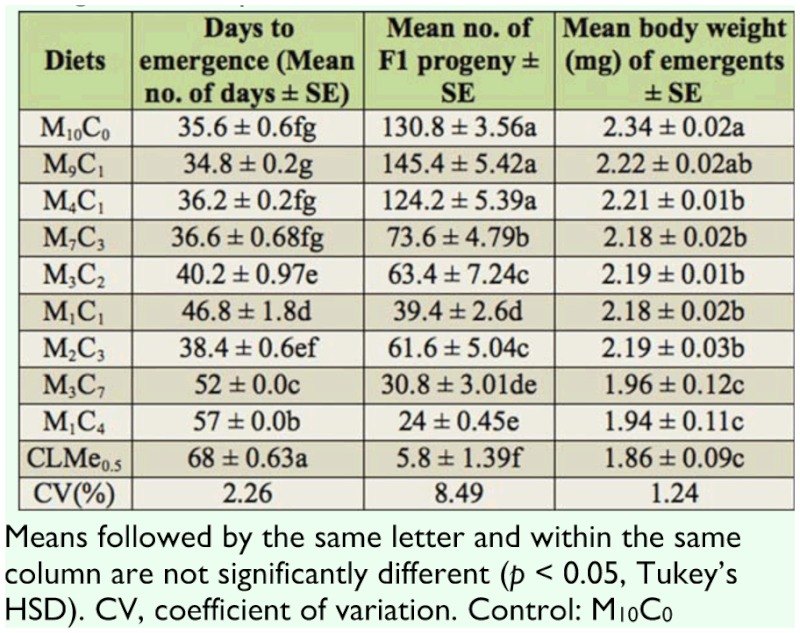
Effect of consumption of AD pellets on F1 emergence of *Sitophilus zeamais.*

**Table 5.**
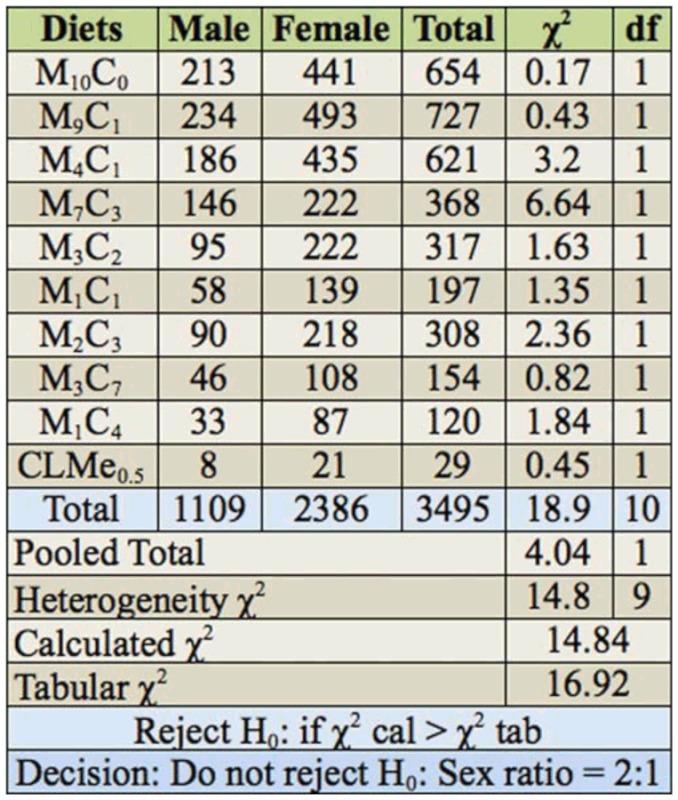
Heterogeneity chi—squared analysis for conformity to 2:1 (female—to—male) sex ratio in *Sitophilus zeamais.*

**Table 6.**
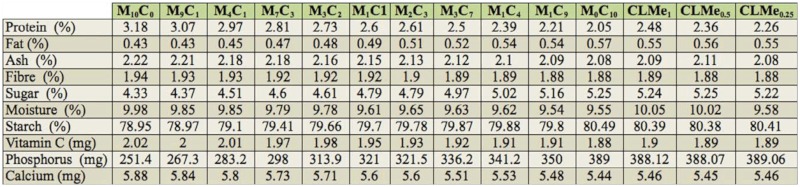
Proximate, mineral, and vitamin composition per 100 g of artificial diet (AD) pellets.
